# Intracranial Electrical Stimulation of the Superior Temporal Gyrus Evokes Rapid Responses in Human Visual Cortex

**DOI:** 10.1111/ejn.70660

**Published:** 2026-08-27

**Authors:** Emily C. Cunningham, David Brang

**Affiliations:** ^1^ Department of Psychology University of Michigan Ann Arbor Michigan USA

**Keywords:** auditory cortex, electrical stimulation, intracranial recordings, visual cortex

## Abstract

Sounds can alter visual processing even at the earliest stages of the cortical pathway. However, our understanding of how acoustic signals are relayed to visual cortex is limited, particularly with respect to the involvement of different regions of auditory cortex in the most rapid stages of this communication across distinct visual areas. Here, we address this question by examining occipital intracranial responses to direct electrical stimulation of the superior temporal gyrus (STG) in 23 patients with epilepsy. Stimulation of mid‐ and posterior‐STG elicited responses in visual cortex as early as 18 ms after onset, with a distribution that favored lateral occipital cortex, lingual gyrus, and anterior calcarine regions near peripheral V1. Early visual cortex (V1/V2) responded most strongly to stimulation over mid‐STG, whereas lateral occipital cortex (near V5/hMT+) exhibited the most robust response to posterior STG stimulation. Responses in visual cortex were organized in a manner similar to that of nonhuman primates, with possible evidence for an exception over the occipital pole. These results support theories that human visual responses to sound are inherited in part from auditory cortex and provide insight into routes through which acoustic information may rapidly facilitate processing of visual input.

## Graphical abstract

Sounds can alter visual processing even at the earliest stages of the cortical pathway, but our understanding of how acoustic signals are communicated to visual cortex is limited. Here, we describe effective connectivity between auditory and visual cortex in humans using corticocortical evoked potentials. Results provide insight into routes supporting interregional information transfer, through which acoustic information may rapidly facilitate processing of visual input.

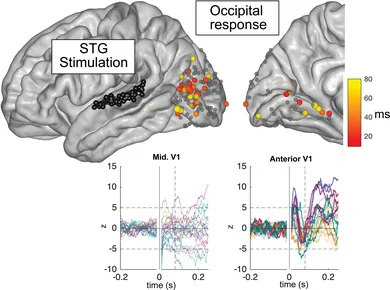

**Abbreviations**
AM: amplitude‑modulated
CCEP: corticocortical evoked potential
CI: confidence interval
DTI: diffusion tensor imaging
EEG: electroencephalography
ERSP: event‑related spectra perturbation
FDR: false discovery rate
HCP‑MMP: Human Connectome Project—Multimodal Parcellation
Hz: Hertz
iEEG: intracranial electroencephalography
lme: linear mixed effects
ms: millisecond
ROI: region of interest
SPES: single‑pulse electrical stimulation
STG: superior temporal gyrus

## 1 Introduction

Visual cortex is increasingly recognized as a site of rapid audiovisual interaction. Even in the absence of concurrent visual stimulation, sounds modulate human visual cortical activity within 100 ms of onset (Brang et al. 2015; Ferraro et al. 2020; Mercier et al. 2013; Plass et al. 2019; Raij et al. 2010), with responses as early as 30 ms recorded in the anterior portion of human V1 (Brang et al. 2015). These responses are thought to indicate a sound‐related alignment of visual cortical activity that enhances sensitivity to subsequent visual input (Brang et al. 2015; Lakatos et al. 2009; Mercier et al. 2013; Naue et al. 2011; Plass et al. 2019; Romei et al. 2012; see also Thorne and Debener 2014). However, relatively little is known regarding the pathways by which these signals are communicated to visual cortex in humans. This information is not only necessary for a complete understanding of how sounds modulate the representation of visual information (e.g., Frassinetti et al. 2002; McDonald et al. 2000; Noesselt et al. 2010; Shams et al. 2000; Vroomen and Gelder 2000) but also augments our understanding of the routes by which visual cortex can be recruited by sounds following visual deprivation.

One persistent question concerns the extent to which these visual responses to sound (especially those emerging before 50 ms) originate from auditory cortex. Nonhuman primate tracing studies point to multiple potential avenues for cross‐sensory communication, including subcortical routes that bypass auditory cortex (possibly through the tectopulvinar system; Lyon et al. 2010), lateral corticocortical pathways between early sensory areas (Falchier et al. 2002; Rockland and Ojima 2003), and polysynaptic routes from auditory cortex through multisensory association areas or thalamic intermediaries (Borra and Rockland 2011; Cappe et al. 2009; Clavagnier et al. 2004). In addition, sounds may exert indirect effects on visual cortical activity by initiating processes associated with arousal, attentional capture, and eye movements.

Given this range of possibilities, it is difficult to isolate contributions of any given pathway using responses to sound alone. Certain features of sound‐evoked visual responses are suggestive. For example, the latency of the response in visual cortex only slightly exceeds that of concurrently recorded responses in auditory cortex (with delays averaging ~30 ms; Raij et al. 2010; but in some cases as brief as ~2 ms; Brang et al. 2015). This latency is consistent with a corticocortical (or corticothalamocortical) relay, but could also arise from subcortical input. Some effort has been made to disambiguate these possibilities by examining responses to amplitude‐modulated (AM) stimuli, based on the observation that whereas subcortical auditory neurons typically entrain to amplitude modulation, a large portion (~20%) of neurons in auditory cortex respond only to onset/offset of an AM stimulus train (Bieser and Müller‐Preuss 1996; see also Harms and Melcher 2002). Consistent with what one would expect of responses inherited from a cortical source, visual cortex is sensitive to sound onset/offset, but does not passively synchronize or entrain to auditory amplitude modulation (Brang et al. 2022; see also Cunningham et al. 2022).

Some comparison can also be drawn between nonhuman primate anatomy and the distribution of human sound‐evoked visual responses. In monkeys, the distribution of projections from auditory to early visual cortex is biased toward the visual periphery (Falchier et al. 2002; Rockland and Ojima 2003), with some projections also identified between superior temporal and visual motion sensitive areas (Majka et al. 2019; Palmer and Rosa 2006). In humans, occipital responses to sound emerge broadly across visual cortex, with some concentration in lateral occipital regions (possibly near human V5/hMT+; Mercier et al. 2013; Plass et al. 2019), and notably fast responses in anterior portions of the calcarine sulcus (Brang et al. 2015; see also Ferraro et al. 2020). Efforts to more directly link nonhuman primate anatomical observations with human sound‐evoked visual responses have been made through noninvasive diffusion‐based imaging (DTI), with at least one report identifying possible connections between primary auditory cortex and both the anterior calcarine sulcus and occipital pole (Beer et al. 2011), and another detailing connections between planum temporale and visual motion‐sensitive hMT+ (Gurtubay‐Antolin et al. 2021). However, limits to the sensitivity of DTI and absence of directional/causal information restrict the conclusions that can be drawn from this approach (particularly given the anticipated sparse distribution of auditory to visual projections).

The aim of this report is to extend these observations by isolating the pattern of directed communication from human auditory to visual cortex. To that end, we take advantage of intracranial EEG (iEEG) recordings made during administration of single‐pulse electrical stimulation (SPES) in patients with epilepsy (open data from van Blooijs et al. 2023). SPES provides an opportunity to estimate effective connectivity between brain regions through the distribution/latency of responses evoked when electrical current is passed between adjacent intracranial contacts. When stimulation and recording sites are located on the cortex, such responses are termed corticocortical evoked potentials, or CCEPs (Matsumoto et al. 2004; for reviews of the technique, see Keller et al. 2014; Matsumoto et al. 2017).

**FIGURE 1:**
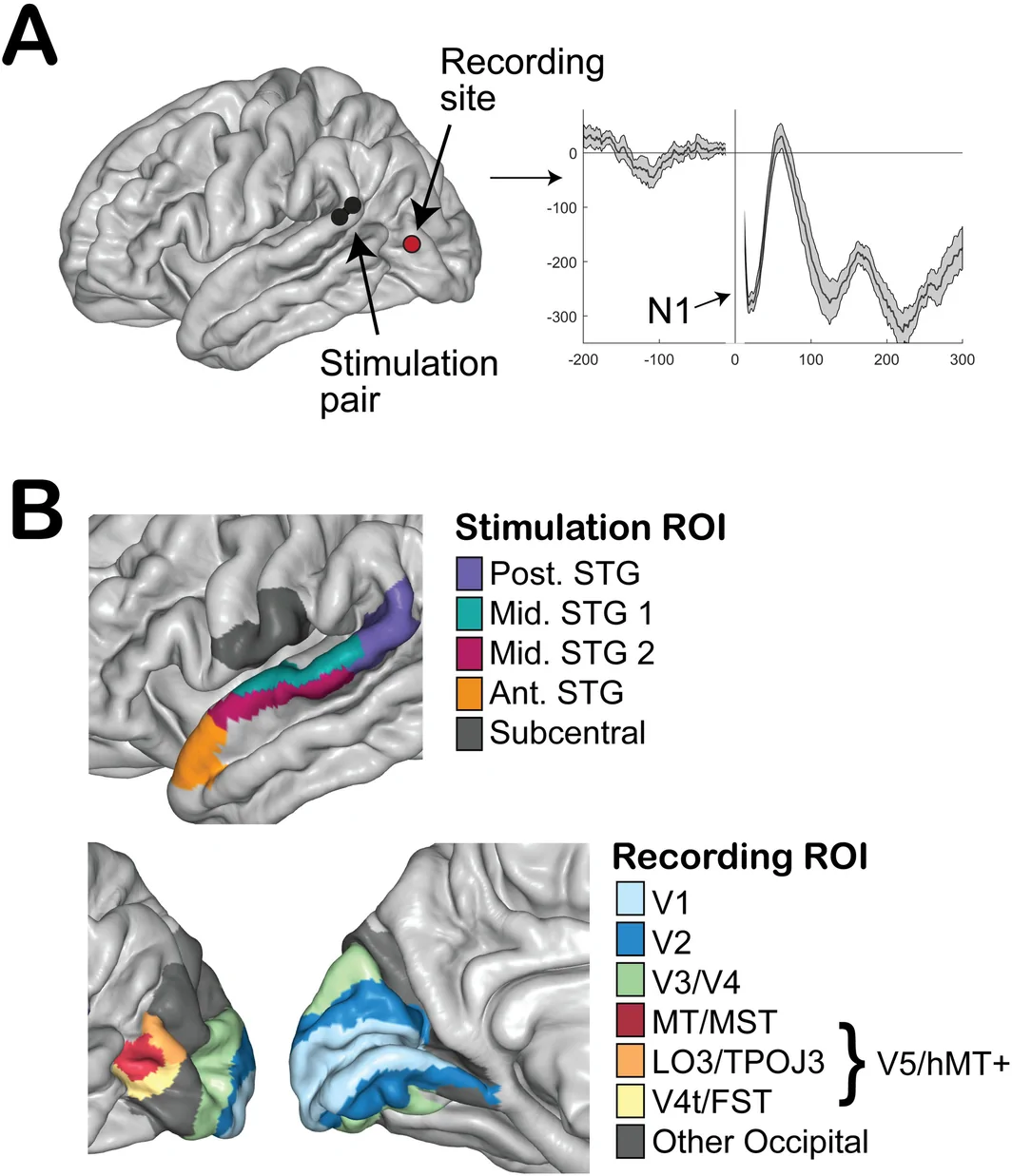
(A) Representative CCEP response with prominent early negative deflection (N1) elicited by stimulation of the posterior STG, and (B) regions of interest in the superior temporal gyrus, subcentral gyrus (control), and occipital lobe illustrated on the Freesurfer average brain.

CCEP waveforms vary in shape, but often display a rapid initial deflection (usually < 50 ms, though longer latencies have been reported, e.g., Araki et al. 2015) along with a subsequent slower wave (~80–250 ms; Figure 1A). The first‐emerging peak in this signal (typically referred to as “N1,” though it may be positive or negative) has been used to estimate transmission speed based on the assumption that it results from orthodromic communication along corticocortical pathways between stimulation and recording sites (a hypothesis supported in part by comparison of CCEPs to responses following stimulation of white matter fiber bundles, Yamao et al. 2014, and by reports that the latency of this peak scales linearly with surface distance from the stimulation site, e.g., Matsumoto et al. 2012; Trebaul et al. 2018; Yamao et al. 2014). The secondary peak, which typically exhibits a larger spatial extent, is thought to reflect either reverberant activity in local corticocortical or corticothalamic circuits following the initial input, or more delayed signal propagated through multisynaptic pathways (see discussion of Matsumoto et al. 2004; corticothalamic investigation by Russo et al. 2025). Additional contributions to the CCEP may arise from cortico‐subcortico‐cortical communication, axonal recruitment, and/or antidromic signal propagation (although this latter explanation is less favored). Although multiple features of the CCEP waveform can provide information regarding interregional communication (e.g., Huang et al. 2023; Miller et al. 2021), here we focus on the presence and latency of the initial “N1” peak as the component of the signal that provides the most insight into the initial relay from stimulation location to recording site (via either corticocortical or corticothalamocortical transmission).

By characterizing the CCEP response in visual cortex after stimulation of regions in and around auditory cortex, our goal is to provide insight into whether rapid visual responses to sound are likely relayed, at least in part, from auditory cortex. Observing low‐latency occipital responses following stimulation of auditory cortex would provide support for this hypothesis, particularly if stimulation‐evoked latencies are comparable to previously estimated delays in the response to sound in both regions. If, on the other hand, the initial visual response to sound largely results from subcortical transmission, the distribution of low‐latency occipital responses should be comparable to what would be expected by chance alone (or to the responses induced by stimulation of a control region like the subcentral gyrus that is not expected to project to early visual cortex; Simonyan and Jürgens 2002, 2003). To be consistent with corticocortical pathways identified in nonhuman primates, occipital N1 responses should additionally be most prevalent and lowest‐latency following stimulation of the mid/posterior portion of the superior temporal gyrus (STG), particularly at recording sites near V5/hMT+ and V1/V2 (and particularly over anterior portions of V1). We therefore targeted these regions for more detailed exploration.

## 2 Method

### 2.1 Participants

Data were drawn from the open repository associated with van Blooijs et al. 2023 (data available at openneuro.org/datasets/ds004080/). A subset (*N* = 23; mean age = 25 years, range 8–51; 11 female/12 male) of the original lifespan sample of 74 patients had combinations of STG stimulation and occipital recording sites that were relevant for the current question (see exclusion/inclusion criteria below), but which were not considered in the initial paper. In all cases, sites were positioned ipsilaterally, with the majority on the left hemisphere (17/23). For the positions of included stimulation pairs and recording sites for each patient, see Figure S1 (additional patient information can be found in Table S1 and in the original repository).

#### 2.1.1 Participant‐Level Exclusion/Inclusion Criteria

Data were first screened to identify patients with combinations of STG and occipital coverage. Patients with relevant coverage were included if they had (a) at least one stimulation pair in which both electrodes were located on the STG, (b) at least one occipital recording location, and (c) at least 10 trials for analysis for at least one relevant stimulation pair.

### 2.2 Data Acquisition

For full details of data acquisition, readers are referred to van Blooijs et al. (2023). In brief, single‐pulse electrical stimulation was conducted as iEEG data were recorded from subdural electrode strips/grids with 1‐cm spacing. Data were initially acquired at a sampling rate of either 512 (*n* = 1) or 2048 Hz (*n* = 22; MicroMed LTM64/128 express device with integrated stimulator). For a given pair of adjacent electrodes, monophasic stimulation (pulse width: 1 ms, current intensity: 8 mA) was typically applied up to 10 times at intervals of 5 s, although in some cases larger numbers of stimulation pulses were recorded.

### 2.3 Processing and Analysis

#### 2.3.1 Selection of Stimulation Pairs and Recording Sites

Labels from the Destrieux et al. (2010) parcellation were used for initial identification of occipital, STG, and subcentral electrodes. The subcentral gyrus was selected as a control stimulation region given its comparable distance to occipital cortex and the expectation of limited communication between subcentral and visual areas (e.g., Simonyan and Jürgens 2002, 2003). For delineation of regions within STG and occipital cortex, we used labels from the multimodal parcellation provided by Glasser et al. (2016; “HCP‐MMP”) mapped to Freesurfer average space. No visual or auditory functional localizer data were available in the dataset; ROI labels therefore reflect anatomical/parcellation assignments rather than independently verified functional responses.

In SPES, electrical current is passed between a pair of electrodes, stimulating the cortex between them. Stimulation pairs were included in the following analyses if both members of a pair were located over the STG, or if one member was located on the STG and the other was located on the inferior portion of the supramarginal gyrus. The region “PSL” from the HCP‐MMP parcellation (Glasser et al. 2016) was used to define the upper border for inclusion of supramarginal electrodes. Pairs that crossed the superior temporal sulcus or lateral fissure were not included (to conservatively isolate stimulation to the STG, as far as possible). Control stimulation pairs were included if they were centered within the subcentral gyrus, excluding pairs that crossed the lateral fissure.

Stimulation pair/recording site combinations were also excluded if they contained any channels marked for exclusion by the criteria described in van Blooijs et al. (2023), or if the total number of trials was less than 10. Note: in a small set of cases (three patients, and 28 CCEPs total) trial counts were between 10 and 20; in all other cases, 10 trials contributed to each CCEP waveform. All trials for a given stimulation pair were included, except in rare instances in which stimulation occurred within 3 s of another trial. Additional exclusion criteria based on data quality are described below.

#### 2.3.2 Preprocessing and Measurement of Evoked Responses

**FIGURE 2:**
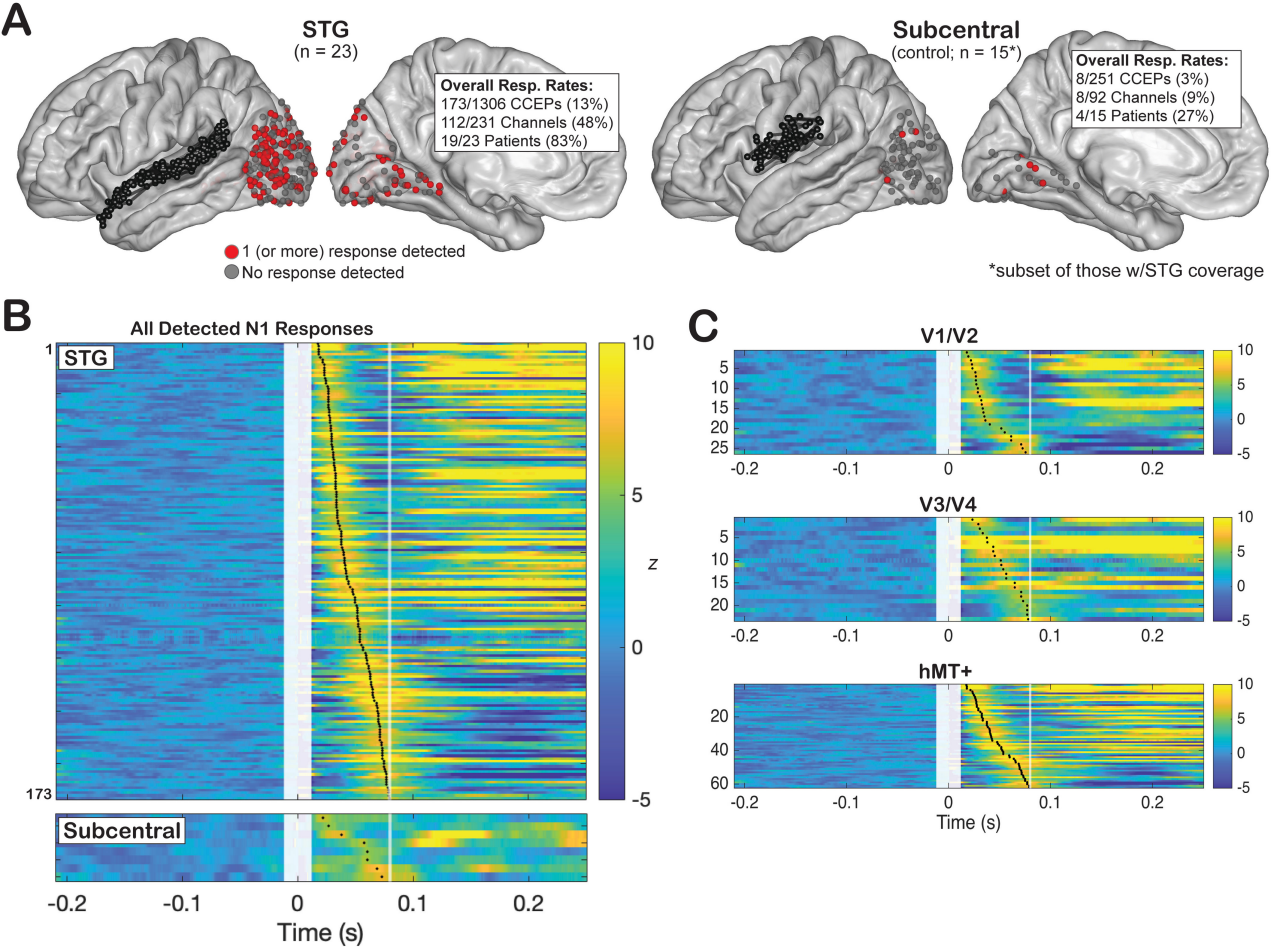
Summary of occipital responses to STG and subcentral stimulation. (A) All stimulation pairs and recording sites for STG and subcentral stimulation. Occipital recording sites are colored according to whether a response was observed at the site (red = at least one response detected; grey = no response detected). Note that the 15 patients with subcentral coverage are a subset of the 23 with STG coverage. (B) Heatmap of all CCEP waveforms for which N1 responses were detected, sorted by latency (upper = STG stimulation; lower = subcentral stimulation). *z*‐scored responses were rectified such that the N1 peak was positive for all waveforms. The white bar surrounding timepoint 0 indicates the stimulation artifact. The white line indicates the cutoff for N1 definition (80 ms). (C) Heatmaps showing responses to STG stimulation for each of the visual regions of interest (subset of responses in B).

As in van Blooijs et al. (2023), no modification of the continuous signal was performed (i.e., data were neither rereferenced from the original mastoid recording reference nor filtered). For measurement of evoked responses, timepoints between −12 and 12 ms were excluded from analysis to limit contamination from stimulation artifacts (for a visualization of the range of the stimulation artifact see Figure S2). CCEPs were evaluated relative to a prestimulus average baseline of [−200 ms to −12 ms]. For peak latency measurement, the averaged response for a given combination of electrode site and stimulation pair was transformed by z‐scoring the waveform relative to the mean and standard deviation over the baseline window. Peaks were identified as the first minimum/maximum in the interval [12–250 ms] with an absolute value exceeding *z* = 5 and a minimum peak prominence of 2 (i.e., peaks were required to have a drop of *z* = 2 on either side before encountering a neighboring peak of greater amplitude). Electrodes were defined as showing a rapid response (N1) if the CCEP waveform contained a detectable peak in the range 12–80 ms following stimulation (see Figures 2 and S3 for visualization of the set of CCEPs, sorted by latency). In the remainder of the document, the term CCEP is used to refer to the averaged waveform for a single stimulation pair/recording site combination.

Saturated responses (defined by post‐stimulation amplitudes exceeding 2000 microvolts) were excluded from analysis (*n* = 3 responses, one patient), as were CCEPs that showed extreme time‐locked line‐noise contamination (defined as having a mean power spectral density over the range 50–60 Hz, prior to stimulation, that was greater than 10× the median value over all stimulation pairs and electrode sites for that patient; *n* = 66 responses over nine patients; this exclusion criterion was data‐driven and implemented to reduce the occurrence of false‐positive “peaks” resulting from line noise). Overall, after these data quality exclusions, 1557 combinations of stimulation pair and recording site (1306 STG stimulation; 251 subcentral stimulation) were considered for analysis (231 total recording sites).

Given that the *z*‐scored waveforms used to compute peak latency are scored relative to variation in the *averaged* waveform over the baseline window, the *z* scores alone contain no information regarding variance in the waveform from trial to trial. To provide a coarse index of stability/variation in amplitude across repeated stimulations, peak latency values were supplemented with Cohen's *d*_*z*_ values computed for peak amplitude (computed by dividing peak amplitude by the standard deviation of the waveform across trials at that timepoint).

#### 2.3.3 Spectral Analyses

Spectral decomposition was performed to characterize the frequency content of the signal over all CCEPs in each region of interest. The intent of this decomposition was descriptive. Trials were epoched from −3 to 3 s to allow a sufficient window for event‐related spectral perturbation (ERSP) computation. The stimulation artifact was removed by interpolating the time‐series between −12 and 12 ms for each trial with piecewise cubic hermite interpolation. Single‐trial spectrograms were computed by convolving the iEEG time series with complex Morlet wavelets (for lower frequencies: 49 center frequencies linearly spaced between 2–50 Hz, with number of cycles per frequency increasing linearly from 3 to 12; for high‐gamma power: 16 center frequencies linearly spaced between 75 and 150 Hz, with number of cycles per frequency increasing linearly from 12 to 24). A time step of ~4 ms was applied to produce time‐frequency plots sampled at 256 Hz. Following convolution, power was extracted as squared amplitude for each trial, timepoint, and frequency. Spectrograms were baseline‐corrected and normalized using the “vssum” approach: for each timepoint the average power over time and trials in the interval [−500 to −200 ms] at that frequency was subtracted, and values were then normalized by dividing by the sum of the value at that timepoint and the associated baseline value. The resulting values are bounded between −1 and 1 and scaled such that ±0.33 indicates a 2× increase/decrease in power from baseline. High‐gamma power values were averaged across all frequencies to produce a single time‐series representation of high frequency activity.

#### 2.3.4 Data Analysis

A nonparametric randomization approach was adopted to provide chance baselines against which to compare the observed data. To generate a baseline that best reflects the expected false‐positive rate in the absence of stimulation, we generated a comparison distribution out of 1000 repetitions of the following: For each CCEP time‐series, we extracted a matched null time‐series centered around a point within the 2 s prior to a trial (selected randomly for each repetition; excluding baseline windows showing saturation or line noise contamination defined by the criteria outlined above). These null time‐series were then processed in the same way as the observed data described above, resulting in 1000 surrogate datasets identical to the main dataset except for the use of null data. In this way, it was possible to evaluate expected false positive rates assuming no effect of stimulation for every observed statistic. Observed values were evaluated against this null distribution, and surrogate *p* values were computed as the proportion of null values matching or exceeding the magnitude of the observed statistic. When observed values exceeded 100% of the null distribution, the surrogate *p* value was recorded as “< 0.001” (the minimum observable *p* value with 1000 datapoints). The 99th percentile of the null distribution was used as a benchmark for evaluating response rates in a given region.

##### 2.3.4.1 Summary Statistics by Stimulation Location and Region

Stimulation pairs over STG were grouped by the HCP‐MMP parcellation label associated with the nearest coordinate in Freesurfer average space to the center of the pair. Stimulation locations with limited representation were combined with neighboring locations to create four summary regions: posterior STG, mid‐STG 1 (PBelt/A4), mid‐STG 2 (A5), and anterior STG (Figure 1B). For region‐of‐interest (ROI) analyses over visual locations, we examined V1/V2, V3/V4, and V5/hMT+ (defined by HCP‐MMP labels). Two of these regions, V1/V2 and hMT+, reflect hypothesized loci of early cross‐modal input (Falchier et al. 2002; Majka et al. 2019; Palmer and Rosa 2006; Rockland and Ojima 2003) and were the subject of detailed examination; V3/V4 was included to provide a point of comparison. Sites were defined as near V5/hMT+ when the nearest coordinate in Freesurfer average space was positioned within the bounds of HCP‐MMP regions MT/MST, TPOJ3/LO3, or V4t/FST (Figure 1B). These regions approximately cover the expected location of human motion‐sensitive cortex (which varies from subject to subject) as defined by multiple atlases (see Huang et al. 2019 for a side‐by‐side visualization).

Patients with right‐ and left‐hemispheric implants were first examined separately to evaluate whether there was any evidence of hemisphere‐specific effects. No effects of hemisphere were identified, and data from both hemispheres were therefore pooled prior to analysis (note, however, that “no effects of hemisphere” should be understood in the context of sample size; we lack power in this sample to perform a detailed analysis of laterality effects).

To avoid pseudoreplication as far as possible, all response rates and latency values (barring initial summary statistics) were reported either in terms of the number of patients showing an effect, the effect per patient (i.e., one value per patient), or in the context of linear mixed effects (lme) models accounting for patient identity (using maximum likelihood estimation; generated using the lme4 package in R; Bates et al. 2015). To provide estimates of uncertainty for fixed effects derived from lme models, confidence intervals (CI) around fixed effects (95%) were computed using the Wald method. To analyze response distribution, response rates were first evaluated against chance *within* each stimulation/recording region combination (using the nonparametric null distribution described above), and then compared across all sets of STG stimulation regions with mixed‐effects models that included a random effect of patient identity and a fixed effect of stimulation location (surrogate *p* values for effects of stimulation location were derived by fitting identical models to the null data and comparing the observed fixed effect against the distribution of null fixed effects; FDR correction was performed across all comparisons using the Benjamini–Hochberg approach). Latencies for each combination of stimulation/recording ROI were summarized (when sufficient data was available) with simple intercept‐only lme models including a random effect of patient identity (i.e., latency ~1 + (1|ID)). The fixed intercept (*β*) of each model and associated 95% CI were used to estimate population mean latency (Bates et al. 2015).

## 3 Results

We examined occipital CCEPs following stimulation of STG in 23 patients, with a focus on the presence and latency of N1 responses occurring within 80 ms of stimulation. Unless otherwise specified, *p* values indicate the proportion of values computed from surrogate data that exceed the observed value. Across 1306 tested STG‐to‐occipital stimulation/recording combinations, 173 CCEPs exhibited a detectable N1 response. Collapsing across stimulation pairs, 112 of 231 unique occipital sites responded to at least one stimulation pair, and at least one responsive site was observed in 19/23 patients (*p* = 0.003; Figures 2 & S3 provide visualization of all waveforms; Figure S4 provides a breakdown of responses by patient for base rate visualization). The average rate of response per patient was 17% (SD = 22%, *p* < 0.001). Responses to subcentral stimulation were comparatively limited, with 8/251 responses detected over 8/92 occipital sites across 4/15 patients (*p* = 0.914). The average response rate to subcentral stimulation per patient was 3% (SD = 8%; *p* = 0.495).

**FIGURE 3:**
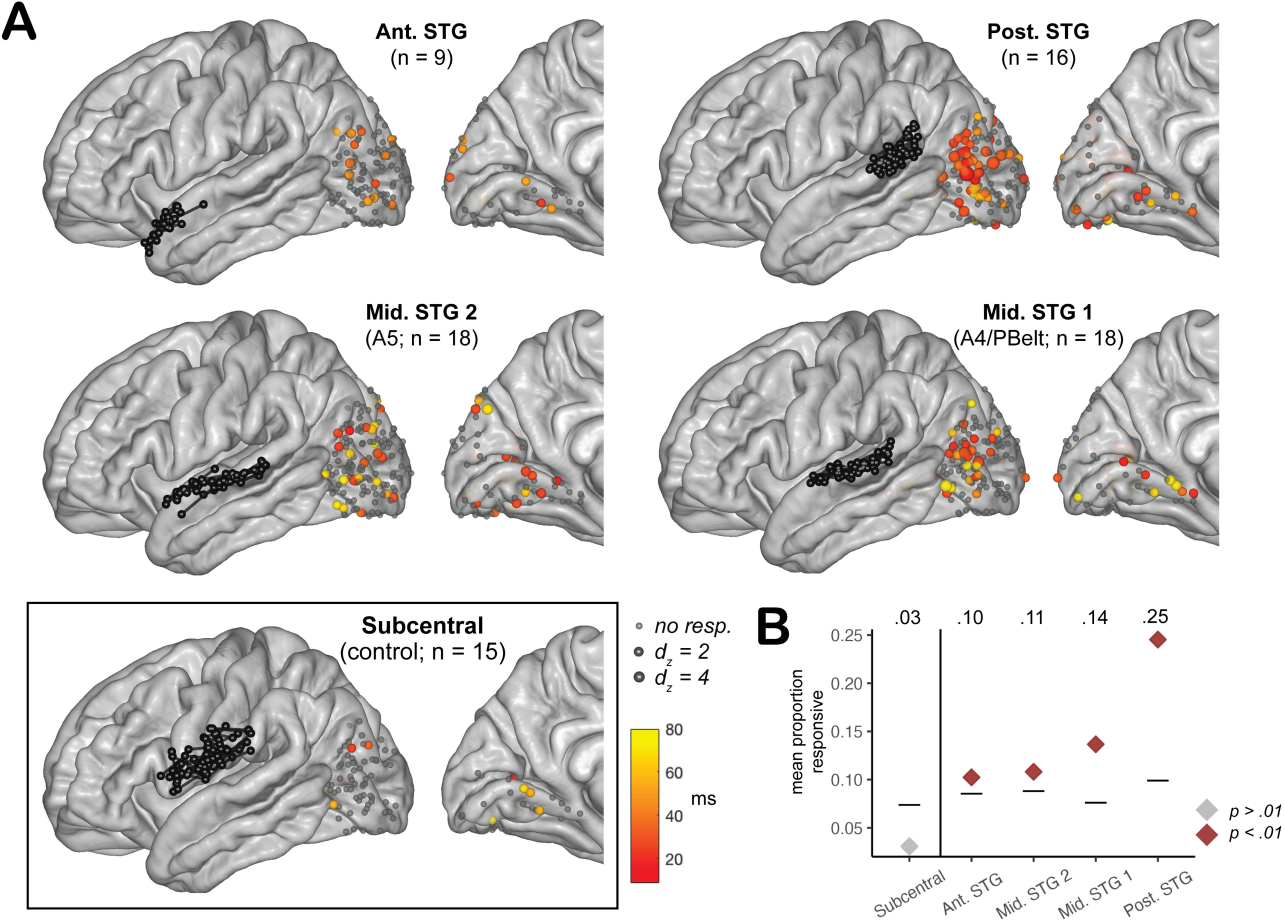
Distribution and latency of occipital responses to STG and Subcentral stimulation. (A) Stimulation pairs and recording sites presented separately for each stimulation ROI. Each recording site is colored according to the fastest detected response at that location (between 12 and 80 ms). Grey indicates no response was detected at that site. The size of each recording site is scaled based on the standardized effect size (Cohen's *d*_*z*_) for the corresponding response. (B) Average response rate across patients for each stimulation ROI. Color indicates whether the response rate exceeded chance baseline (99th percentile) derived from null data. Black lines indicate the 99th percentile of the null distribution.

Figure 3 summarizes occipital N1 responses as a function of stimulation location along the STG. The average response rate across patients was greatest following stimulation of posterior STG and declined with increasingly anterior stimulation (Figure 3B; Average response rates and surrogate *p* values: Anterior STG: M_resp_rate_ = 10%, *p* = 0.003; mid‐STG 2: M_resp_rate_ = 11%, *p* = 0.001; mid‐STG 1: M_resp_rate_ = 14%, *p* < 0.001; Posterior STG: M_resp_rate_ = 25%, *p* < 0.001; compare with subcentral response rate above). Qualitatively, N1 responses to posterior STG stimulation tended to concentrate around lateral occipital cortex (nearest in proximity to the stimulation site), becoming more diffuse (and longer‐latency) with distance as stimulation approached anterior STG (Figure 3A).

**FIGURE 4:**
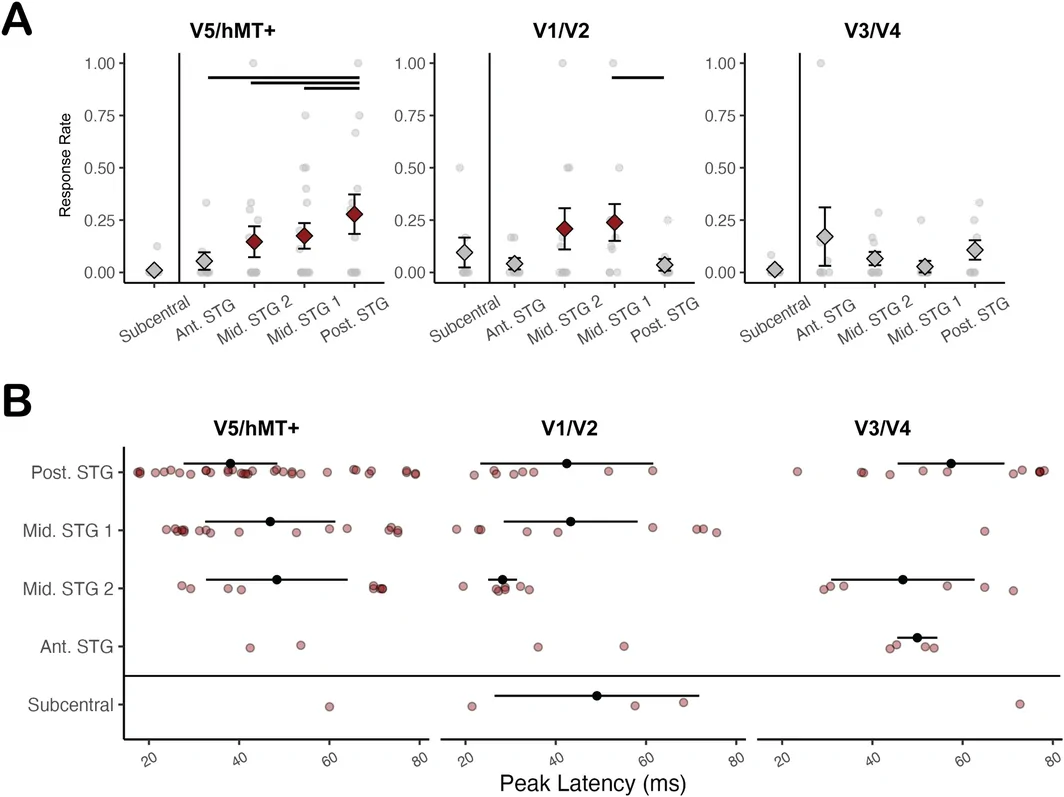
Response rates and latencies for each occipital ROI. (A) Response rates by stimulation location. Each grey dot indicates the proportion of responsive CCEPs relative to the total number of CCEP waveforms for a single patient. Diamonds indicate the average response rate across patients (error bars = SEM). Red diamonds indicate situations in which the average response rate exceeded the 99th percentile of the associated chance baseline (as in Figure 3). Horizontal bars indicate groups for which differences in response rates were greater than expected given the chance distribution derived from null data (after FDR correction). (B) Latencies for all detected N1 responses (red points). Estimates of mean latency and associated 95% CIs derived from LME models are plotted with black points/bars (where sufficient data was available).

The distributions of response rates and N1 latencies as a function of stimulation location and occipital ROI are reported in Figure 4. Within V5/hMT+, both mid‐ and posterior‐STG stimulation yielded above‐chance response rates (Figure 4A). The proportion of detected responses per patient increased with proximity as stimulation moved from anterior (5%) to posterior STG (28%; Figure 4A; FDR‐corrected *p* < 0.05 for all contrasts that included Posterior STG; all other contrasts *n.s*.). Within V1/V2, response rates only exceeded chance levels following mid‐STG stimulation (i.e., when stimulation was centered over likely auditory association areas accessible to contacts placed on the outer surface of mid‐STG), and declined with more anterior/posterior stimulation (Figure 4A; note that only the reduction in response rate between region “mid‐STG 1” and Posterior STG was robust to FDR correction; all other contrasts *n.s*.). This effect appears largely driven by responses in anterior portions of the calcarine sulcus and lingual gyrus (Figure 3A). Within V3/V4, response rates did not show clear differentiation by stimulation location.

Latencies of all detected N1 responses are presented by ROI in Figure 4B and summarized in Table 1 (along with 95% CIs and latencies of the fastest observed response in each case). Given that there were pronounced differences in response rate as a function of stimulation location, the following description of latency distributions should be interpreted with caution. Within V5/hMT+, the distribution of responses to mid‐STG stimulation (both regions 1 & 2) was somewhat bimodal, with most N1 peaks hovering between ~25 and 40 ms, and a few outlying responses between 70–80 ms, resulting in overall latency estimates of *β* = 47–48 ms. Responses to posterior STG stimulation were also broadly distributed, but with a somewhat greater concentration of low‐latency responses (< 40 ms), resulting in an estimated overall latency of *β* = 38 ms (10 ms faster on average than mid‐STG stimulation; Figure 4B; Table 1). The fastest overall response in V5/hMT+ (18 ms) was also recorded following stimulation of posterior STG. Anterior STG and subcentral stimulation yielded too few suprathreshold responses to derive reasonable estimates of latency; All responses that were detected exceeded 40 ms.

**TABLE 1:** N1 latency estimates by stimulation and recording ROI.

Recording ROI	Stimulation ROI	*β* (ms)	95% CI (ms)	Min. latency (ms)
hMT+	Subcentral	NA	NA	60
hMT+	Ant. STG	NA	NA	42
hMT+	Mid. STG 2	48	[33, 64]	27
hMT+	Mid. STG 1	47	[32, 61]	24
hMT+	Posterior STG	38	[28, 48]	18
V1/V2	Subcentral	49	[26, 72]	21
V1/V2	Ant. STG	NA	NA	36
V1/V2	Mid. STG 2	28	[25, 31]	19
V1/V2	Mid. STG 1	43	[28, 58]	18
V1/V2	Posterior STG	42	[23, 62]	22
V3/V4	Subcentral	NA	NA	73
V3/V4	Ant. STG	50	[46, 54]	44
V3/V4	Mid. STG 2	47	[31, 63]	29
V3/V4	Mid. STG 1	NA	NA	65
V3/V4	Posterior STG	57	[46, 69]	23

Within V1/V2, there were enough detected N1 responses to derive latency estimates for posterior STG and both mid‐STG locations. When stimulation occurred over posterior STG or neighboring mid‐STG 1, the distribution of latencies was fairly broad, producing latency estimates of *β =* 42–43 ms (although a number of peaks emerged prior to 40 ms). In contrast, all responses to stimulation of the more anterior mid‐STG 2 ROI emerged before 40 ms (for an overall latency estimate of *β* = 28 ms). The fastest responses in V1/V2 (18–19 ms) emerged following stimulation of both mid‐STG regions. As with V5/hMT+, anterior STG stimulation produced too few detectable responses to derive a latency estimate (and the responses that were detected were comparatively slow; Figure 4B). Subcentral stimulation also yielded few detectable responses, with latencies that were broadly distributed.

Within V3/V4, response latencies were heterogeneous. Although some low‐latency responses were observed, latency estimates across all stimulation regions hovered between *β =* 47–57 ms.

Given that these data were derived from a lifespan investigation of age‐related changes in transmission speed, a concern might be raised regarding the possibility of interactions between patient age and latency estimates. Consistent with van Blooijs et al. (2023), there was a negative relation between patient age (8–51 years) and overall N1 latency (after averaging across occipital sites and stimulation regions) in this dataset (*ρ*_spearman_ = −0.53, *p* = 0.030; Figure S5). However, inspection of age‐related effects as a function of stimulation region/recording site did not suggest that any of the results reported above were attributable to age‐related confounds (although it should be noted that there was insufficient statistical power in this dataset to provide a detailed breakdown of age‐related effects by subregion).

The spectral content of CCEPs was qualitatively similar from region to region (Figures S6 and S7). In both V5/hMT+ and V1/V2, CCEPs were largely dominated by low‐frequency activity (< 30 Hz), with no evidence of stimulation‐evoked activity in the high‐gamma range. This pattern is consistent with signal propagation of a feedback type (Bastos et al. 2015; Buffalo et al. 2011), without the high‐frequency activity often taken as an indicator of local spiking (Manning et al. 2009).

### 3.1 Exploratory Analyses of V1 ROI

**FIGURE 5:**
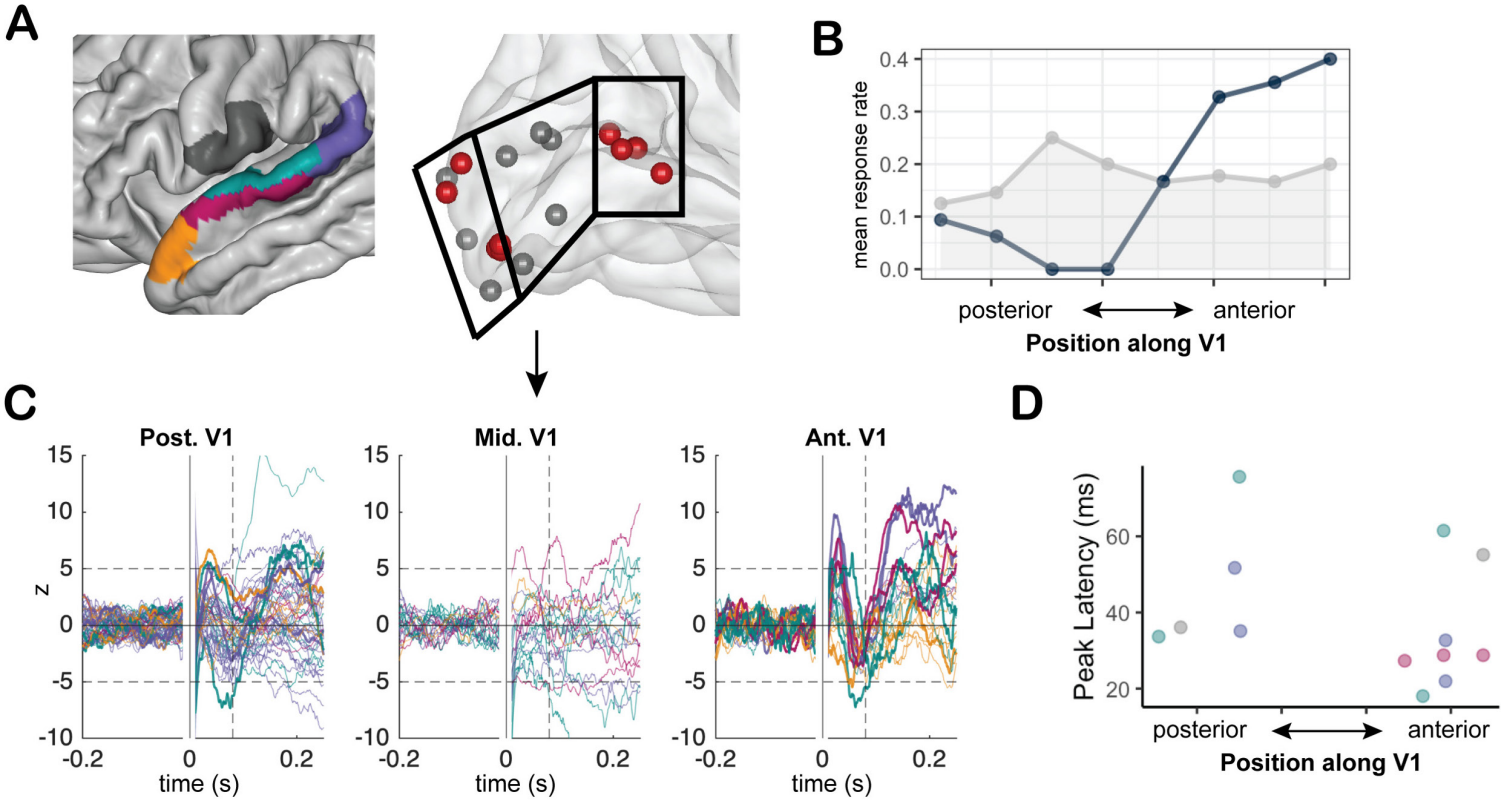
V1 responses by eccentricity. (A) Summary of responsive (red) and nonresponsive (grey) V1 sites. (B) Average response rate as a function of position along V1 (from posterior to anterior). Dark line indicates observed response rate; Grey shaded region indicates 99th percentile of the permutation distribution at each eccentricity. (C) All CCEP waveforms, colored according to stimulation region as shown in A, and split by location along V1. Dashed vertical/horizontal lines indicate the thresholds for response detection. (D) Latencies of all observed N1 responses, plotted by position along V1. Color indicates stimulation location by the key shown in A.

**FIGURE 6:**
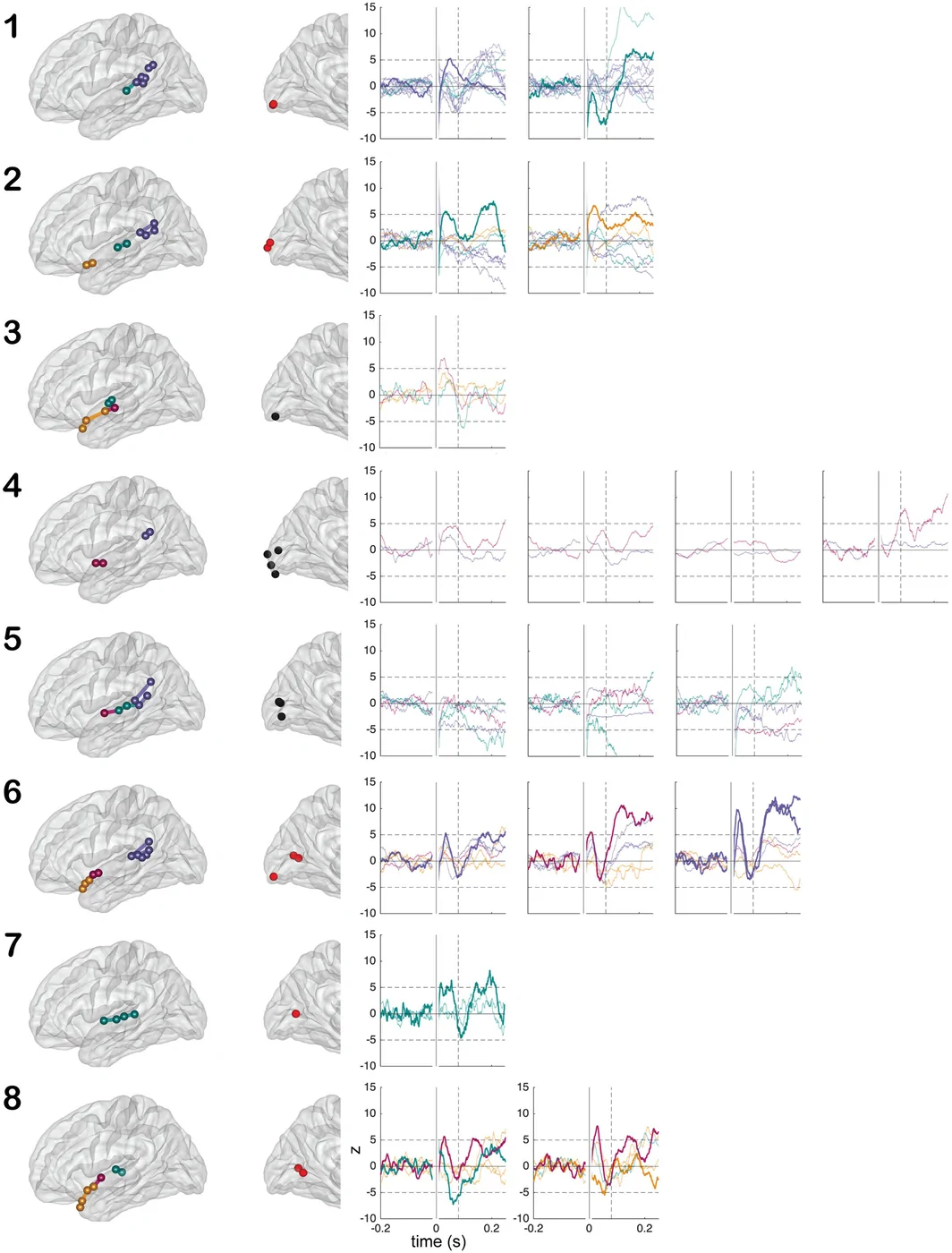
CCEP waveforms for all patients with V1 recording sites. Each row contains data for a single patient, ordered from top to bottom according to approximate location of recording sites along V1. Stimulation pairs are colored according to stimulation region. Recording sites are colored according to whether a response was detected at that site. Each waveform plot contains data from a single recording site (with waveforms colored according to stimulation region). Waveform plots for each patient are arranged left‐to‐right by location of the recording site (anterior–posterior). Waveforms with detected N1 responses are plotted in “bold.”

In nonhuman primates, auditory projections preferentially target anterior regions of V1 corresponding to representation of the peripheral visual field (Falchier et al. 2002; Rockland and Ojima 2003). In this dataset, electrode coverage of multiple locations along V1 provided a rare opportunity to explore the presence of a similar bias in the human CCEP. To this end, we examined response rates and latencies as a function of the position of V1 recording sites along the anterior–posterior axis (coarsely grouped into anterior/mid/posterior locations by Freesurfer average y‐coordinate). As shown in Figures 5 and 6, responses were detected in the most anterior portion of the calcarine sulcus (with an average detection rate of 33% across three patients with anterior calcarine sites), and to some extent the occipital pole (9% across four patients; below the corresponding chance threshold). There were no detectable N1 responses in mid‐calcarine locations (Figure 5; Note: to supplement the grouped data, we also performed a sliding‐window assessment of response rate, grouping CCEPs by y‐coordinate with a step of 5 and a window size of 10; Figure 5B). The latency of anterior calcarine responses ranged from 18 to 61 ms (Figure 5D), with 5/8 peaks emerging within 30 ms of stimulation (*β* = 33 ms, 95% CI [22, 45]). The latency of occipital polar responses ranged from 34 to 76 ms (*β* = 45 ms, 95% CI [29, 62]).

### 3.2 Exploratory Analyses of V5/hMT+ ROI

**FIGURE 7:**
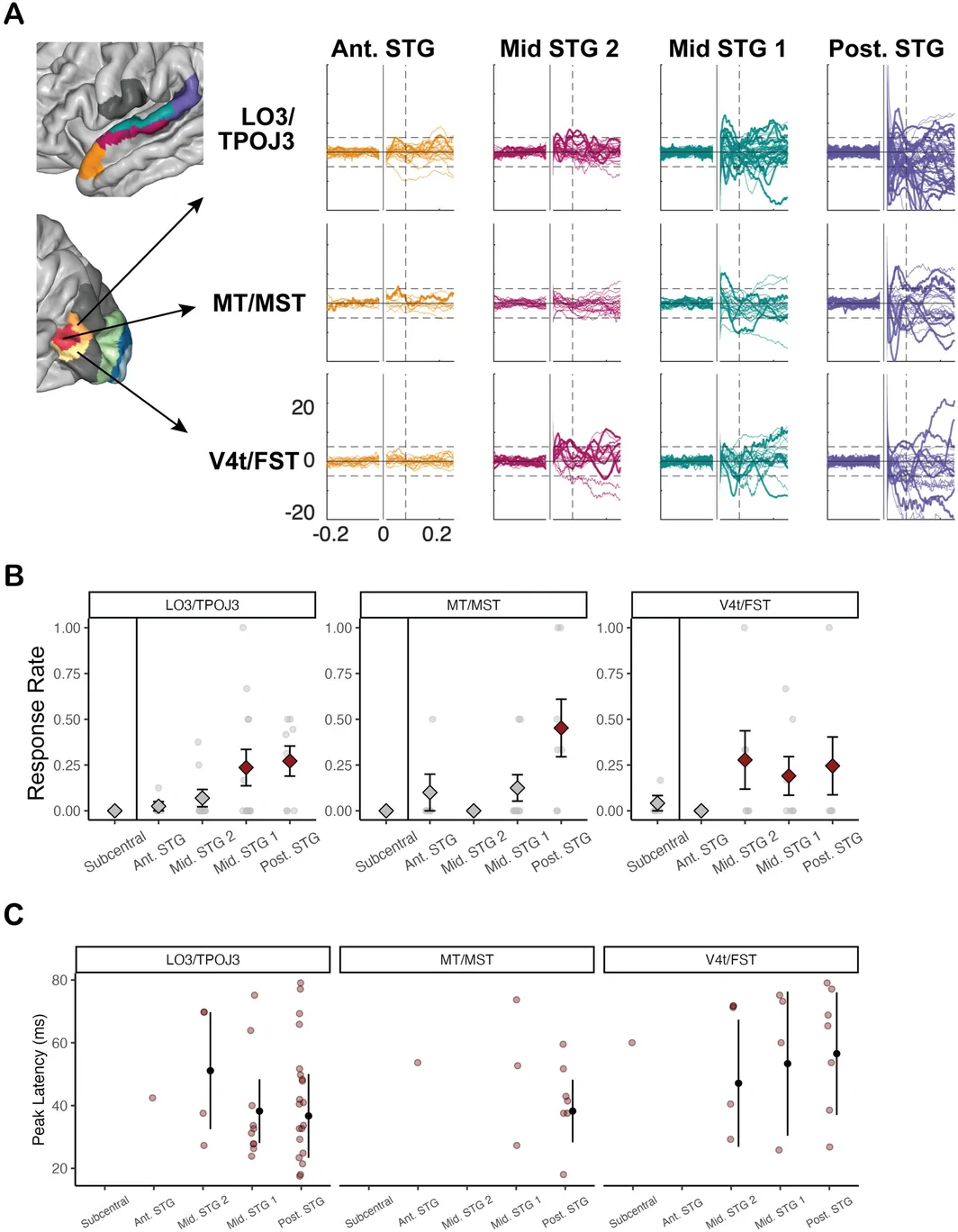
Summary of hMT+ responses by subregion. (A) All CCEPs for all three subregions of the greater V5/hMT+ ROI, separated by stimulation location along STG. Dashed vertical/horizontal lines indicate thresholds for N1 evaluation. (B) Average response rates for each subregion, plotted in the same manner as Figure 4. (C) Response latencies for all N1 responses in each subregion, plotted in the same manner as Figure 4.

Reports of sound‐evoked visual activity (Plass et al. 2019) and human/nonhuman primate connectivity (Gurtubay‐Antolin et al. 2021; Majka et al. 2019; Palmer and Rosa 2006) also highlight V5/hMT+ as a likely site of early cross‐modal input, particularly from auditory motion‐sensitive areas along the planum temporale. This is consistent with the observation of posterior‐STG‐dominant responses in the broad V5/hMT+ ROI (Figures 3A and 4A). To clarify the specificity of responses within this region, we examined CCEPs within three subregions defining the greater ROI (MT/MST, the more dorsal area covered by LO3/TPOJ3, and the more ventral area V4t/FST). Although there were more detected responses overall in sites located within LO3/TPOJ3, the response to posterior STG stimulation was maintained across all three locations (Figure 7). Notably, within MT/MST, responses to stimulation of other locations along the STG were comparatively limited (i.e., responses in MT/MST showed some selectivity to posterior STG; Figure 7B). With respect to latency, estimates in MT/MST and LO3/TPOJ3 following stimulation of posterior STG were roughly comparable (*β*s = 37–38 ms; though the distribution of responses in LO3/TPOJ3 was denser, with a larger number of low‐latency responses to both mid‐ and posterior STG stimulation; Figure 7C). Responses in V4t/FST tended to emerge later (*β*_post_STG_ = 57 ms), consistent with a dorsal bias in communication from auditory areas (see Table S2 for a full summary of latency estimates by stimulation location for each hMT+ subregion).

## 4 Discussion

An accumulating body of evidence indirectly supports the rapid transmission of information from auditory to visual cortex in humans. Here, our goal was to extend these observations by isolating and characterizing effective connectivity between superior temporal and occipital cortex in the context of intracranial recordings during single‐pulse electrical stimulation in 23 patients with epilepsy (van Blooijs et al. 2023).

Our first aim was to determine whether communication from auditory to visual cortex occurs with sufficient speed to contribute to the initial visual response to sound. Here, V1/V2 responded as early as 18 ms to electrical stimulation over mid‐ and posterior STG, with an average latency of between 28 and 43 ms depending on the stimulated region. This range of latencies is broadly compatible with previous estimates that place the average delay between the initial response to sound in human auditory versus visual cortex at between 30 and 35 ms (MEG; Raij et al. 2010), particularly given differences in measurement strategy across studies. That is, the approach here is based on identification of peaks in the signal, whereas previous investigations of sound‐evoked patterns have typically focused on the earliest deviation from baseline. In CCEP contexts, the initial deviation from baseline can be difficult to determine given that it may be confounded to a degree with the stimulation artifact, but when reported, N1 onset estimates tend to precede peak latency estimates by at least 10 ms (e.g., Trebaul et al. 2018). Adjusting peak latency estimates downward by ~10 ms would place many of the observations in this study within approximately the same latency range as the estimated delay in sound‐evoked onset between auditory and visual cortex.

One might ask whether the N1 latencies observed here can reasonably account for the most rapid reports of sound‐evoked visual responses. For example, responses to sound have been reported as early as 28 ms in anterior calcarine sulcus (Brang et al. 2015). In that report, the earliest concurrent response recorded in STG emerged at approximately 26 ms (i.e., an offset of ~2 ms between cortices). However, this estimate was derived from depth electrodes located on the inferior bank of the STG within superior temporal sulcus. Direct recordings from primary auditory areas in humans suggest that initial cortical responses typically emerge between 8 and 13 ms (Liégeois‐Chauvel et al. 1991), for an estimated offset of 15–20 ms between the earliest responses recorded in auditory and visual areas. This latency is compatible with the fastest stimulation‐evoked responses we observed (18 ms). The limited number of observed responses in this latency range may stem from limitations in coverage (in that coverage was restricted to the lateral surface of the STG, limiting our capacity to measure responses to stimulation of primary auditory cortex). However, it is also possible that such rapid responses do not recruit auditory cortex, but are instead generated via subcortical projections (possibly from superior colliculus/pulvinar to visual cortex; e.g., Lyon et al. 2010).

Although functional localizers were not available for these patients, the distribution of responses in this study also corresponds well to previous reports of the distribution of sound‐evoked responses in human occipital cortex (Plass et al. 2019; Mercier et al. 2013; Brang et al. 2015; Ferraro et al. 2020). That is, N1 responses were most prevalent in lateral occipital cortex and showed a degree of bias toward the anterior portion of V1. The current dataset contained only ipsilateral stimulation/recording combinations, but there is reason to expect that cross‐modal communication between early sensory cortices is restricted to the same hemisphere, given animal work and prior research in humans suggesting primacy for spatially aligned auditory–visual stimuli (McDonald et al. 2000; Plass et al. 2019; Wallace et al. 1996).

An additional aim of this work was to evaluate the correspondence between stimulation‐evoked responses and anticipated patterns of auditory‐to‐visual corticocortical connectivity derived from nonhuman primate tracing studies and human DTI. However, it should be noted that although N1 responses in CCEP waveforms are often assumed to reflect corticocortical communication, CCEP responses alone cannot fully adjudicate between monosynaptic and polysynaptic communication (i.e., during in vitro stimulation of cortical slices, initial short‐range responses typically emerge within 10 ms; in the CCEP, long‐range responses of 50 ms or more afford sufficient time for polysynaptic propagation). In addition, risks of axonal recruitment or antidromic propagation complicate interpretation. The correspondence (or lack thereof) across species/methodologies is therefore suggestive, but not conclusive.

The distribution of CCEP responses conformed to expectations derived from anatomical/structural work in several ways. First, the greater prevalence of responses to mid/posterior STG stimulation (although it follows naturally from the distance gradient along STG), was consistent with expectations of communication from caudal belt and planum temporale to visual cortex (Falchier et al. 2002; Rockland and Ojima 2003; Majka et al. 2019; Gurtubay‐Antolin et al. 2021). The corresponding lack of strong, low‐latency responses to stimulation of the most anterior portions of the STG was perhaps unsurprising, given limited precedent. Some confidence that these results were not an artifact of distance gradient alone was provided by stimulation of a control region in subcentral gyrus, which, though similar in surface distance to mid‐STG sites, elicited few occipital responses.

In addition, animal studies suggest that auditory inputs to visual cortical neurons have a feedback‐type laminar profile (Falchier et al. 2012) and exert a modulatory influence on visual cortical processing (Allman and Meredith 2007; Meredith et al. 2009; Wang et al. 2008). That is, rather than directly eliciting action potentials in visual cortex, auditory inputs may instead produce subthreshold changes in membrane potential, resulting in a phase‐reset of ongoing oscillatory activity that facilitates the response to upcoming visual input (Mercier et al. 2013; Thorne and Debener 2014). The spectral profiles of occipital CCEPs were generally consistent with this type of effect. Superior temporal stimulation produced low‐frequency modulation (< 30 Hz; more typical of feedback signal propagation), without inducing higher frequency oscillatory activity (~40–60 Hz) typically associated with feedforward signal propagation (e.g., Bastos et al. 2015; Buffalo et al. 2011). We also did not see evidence of the high‐frequency broadband response often associated with neuronal spiking (Manning et al. 2009). This observation is inconsistent with at least one previous report identifying high‐gamma responses to sound in visual cortex (Ferraro et al. 2020). This inconsistency could be superficially attributable to differences in contact position or measurement approach, but might again hint at sources for the visual response to sound that are not captured by the current study (e.g., subcortical sources or auditory cortical sources inaccessible to surface stimulation).

Finally, the peripheral bias in V1 response is consistent with reports of increased density of auditory‐to‐visual projections with eccentricity in nonhuman primates (Falchier et al. 2002; Rockland and Ojima 2003), and the lateral occipital response to posterior STG stimulation maps well to proposed connections between planum temporale and hMT+ (e.g., Gurtubay‐Antolin et al. 2021). Responses in these regions are discussed in more detail in the following sections.

### 4.1 V1 Responses

As summarized above, within anatomically defined V1, superior temporal stimulation evoked particularly strong N1 responses at anterior electrode sites. These anterior calcarine responses were not constrained to a single stimulation region, but emerged following stimulation of both middle and posterior portions of the STG. The distribution of effects within the middle portion of the STG was somewhat counterintuitive. Based on the nonhuman primate literature, in which auditory projections to peripheral V1 are most prevalent from the caudal belt and parabelt (Falchier et al. 2002; Rockland and Ojima 2003), we expected to see strong anterior visual responses with stimulation between the transverse gyrus and planum temporale (i.e., when contacts were placed along the posterior half of the STG, as in Figure 6, row 7). However, this was not the case, at least in our limited sample. V1 responses to stimulation of this region were relatively limited, and did not conform to the stereotypical CCEP N1/N2 shape. For example, in at least two cases (Figure 6, rows 2 and 7, green waveforms), the response was almost periodic, with wide, rounded peaks rather than sharp initial deflections. Stimulation just anterior to the transverse gyrus yielded faster and more stereotypically shaped initial peaks (Figure 6).

Interpretation of these observations relative to the literature is complicated not only by the sample size, but also by the placement of electrodes on the cortical surface in this dataset. As previously noted, this placement limits access to core and belt auditory regions embedded in Heschl's gyrus, such that we can make only limited assertions regarding stimulation of early auditory cortex. To more completely describe communication between primary sensory cortices (and to better understand the results above), it will be necessary to assemble data in which stimulation is applied over contacts adjacent to auditory belt/parabelt regions where many of the visual projections identified in macaques originate (Falchier et al. 2002, 2012; Rockland and Ojima 2003).

STG stimulation also evoked possible responses over the occipital pole, although there were no detectable responses in mid‐calcarine sites. Occipital polar responses were generally weaker than those over the anterior calcarine sulcus (see Figure 6, subject 6), and they have limited support in nonhuman primate data, where the rate of auditory projections to V1 declines monotonically with decreasing visual eccentricity (approaching zero near the center of the visual field; Falchier et al. 2002). At least two intracranial reports in humans also describe limited, if any, response to sound in posterior occipital contacts (Mercier et al. 2013; Plass et al. 2019), with similar eccentricity effects reported in the context of functional neuroimaging (Vetter et al. 2020). However, occipital polar responses are compatible with results from human DTI that identify possible connections between auditory areas and both anterior and polar portions of early visual cortex (Beer et al. 2011), as well as cross‐modal effects reported in the context of transcranial magnetic stimulation at the occipital pole (Romei et al. 2007, 2009), and could point to an asymmetric, bimodal distribution of projections in humans that diverges from that observed in nonhuman primates. Given that few patients had coverage of multiple locations within V1 in this sample, additional work will be necessary to thoroughly address this question.

The observation of responses near the occipital pole may also be consistent with reports that have used noninvasive scalp EEG to examine cross‐sensory interactions. For example, combining an auditory stimulus with a visual stimulus will produce modulations of the parieto‐occipital evoked response approximately 40–90 ms after stimulation onset (Giard and Peronnet 1999; Mishra et al. 2007; Molholm et al. 2002). This response has been linked to modulation of the C1 component (Molholm et al. 2002) thought to originate in striate cortex. It is difficult to imagine that a modulation of the C1 component observable at the scalp could arise from auditory inputs deep within the calcarine fissure. However, relays from auditory cortex to posterior calcarine areas (with a latency of ~45 ms) could plausibly yield such an interaction.

Notably, these noninvasive studies also report an asymmetry in modulation strength, with a bias toward the right hemisphere (Giard and Peronnet 1999; Molholm et al. 2002). In our initial assessment of laterality effects (prior to pooling data across hemispheres), we did not observe differences as a function of hemisphere of stimulation. However, we also did not have sufficient statistical power in this sample to perform an extensive comparison of effects of stimulation across hemispheres, nor did we have any patients in this sample with concurrent recording in both hemispheres (which would allow within‐patient investigation of asymmetric organization). The question of hemispheric asymmetry, like the question of contralateral versus ipsilateral communication, remains an open avenue for inquiry.

### 4.2 V5/hMT+ Responses

The most pronounced responses to stimulation emerged over lateral occipital cortex, particularly after stimulation of posterior STG. Although this pattern is not entirely surprising, given that lateral occipital cortex is closest in proximity to the sites of stimulation, it is consistent with at least one prior report detailing the distribution of sound‐evoked visual responses across human visual cortex (Plass et al. 2019). As suggested by Plass et al. (2019), these lateral clusters of responses could reflect communication from auditory cortex/planum temporale to visual motion‐sensitive area V5/hMT+. There is some structural evidence in both nonhuman primates and humans for the existence of projections between these areas (NHP: Majka et al. 2019; Palmer and Rosa 2006; DTI: Gurtubay‐Antolin et al. 2021). These observations are paralleled by functional reports that describe recruitment of visual motion‐sensitive cortex in response to auditory motion in the early blind (Dormal et al. 2016; Poirier et al. 2006; Strnad et al. 2013; Bedny et al. 2010).

Although motion‐based localizers were not available in this study, responses within anatomically defined V5/hMT+ were broadly compatible with the proposal of a relay conveying auditory positional or motion‐related information from posterior STG to visual motion‐sensitive cortex. Within V5/hMT+, responses were generally strongest following posterior STG stimulation, with a dorsal bias such that sites over MT/MST and neighboring LO3/TPOJ3 responded faster and more frequently than sites over V4t/FST. The earliest CCEP responses in MT/MST and LO3/TPOJ3 emerged ~18 ms following posterior STG stimulation, and the average latency of responses was approximately 38 ms. To our knowledge, there are no existing estimates of relative latencies derived from concurrent recordings in planum temporale and hMT+ during auditory stimulation. However, in lateral occipital cortex, responses to sound tend to onset ~50–100 ms following stimulation (Plass et al. 2019). In the planum temporale, initial auditory evoked potentials emerge around 30 ms (Liégeois‐Chauvel et al. 1994). Taken together, these reports suggest an approximate delay between initial sound‐evoked responses in both regions that is compatible with the latencies of the earliest CCEP N1 peaks, although this is an area that would benefit from concurrent recordings directly comparing CCEP latencies with delays in sound‐evoked response.

### 4.3 Conclusions

Overall, these results support the hypothesis that the initial response to sound in human visual cortex originates, at least in part, from auditory cortex. In addition, the results suggest that rapid communication from auditory to visual cortex in humans is organized in a manner similar (though perhaps not identical) to that of nonhuman primates. Notably, although these results are compatible with the possibility of corticocortical intersensory communication, they cannot be used to adjudicate contributions of other pathways to the sound‐evoked visual response (including subcortical, corticothalamocortical, and/or pathways through higher order multisensory cortex). The relative contributions of these pathways remain an open avenue for inquiry. Although the current data was obtained from patients with epilepsy, the compatibility of these observations with reports of sound‐evoked responses in other patients (Brang et al. 2015; Ferraro et al. 2020; Mercier et al. 2013; Plass et al. 2019), connectivity in nonpatient samples (Beer et al. 2011; Gurtubay‐Antolin et al. 2021), and tractography in nonhuman primates (Falchier et al. 2012; Majka et al. 2019; Palmer and Rosa 2006; Rockland and Ojima 2003) provides a measure of confidence in the results.

## Author Contributions

**Emily C. Cunningham:** conceptualization, methodology, writing – original draft, writing – review and editing, visualization. **David Brang:** conceptualization, methodology, writing – review and editing, visualization, supervision.

## Funding

This project was partially supported by National Institutes of Health NIDCD R01 DC020717.

## Ethics Statement

This study entails secondary analyses of publicly available, de‐identified data. No new data were collected from humans or animals. The research qualifies for exemption under the U.S. Common Rule. No additional institutional review was required.

## Conflicts of Interest

The authors declare no conflicts of interest.

## Supporting information


**Table S1:** Patient details.
**Table S2:** Latency estimates by stimulation and recording ‘subregion’ within V5/hMT+ ROI.
**Figure S1: Included sites and stimulation pairs by patient.** IDs correspond to index in original repository. Locations are displayed on the fsaverage brain. Lines link stimulation pairs. No patients had contralateral combinations of stimulation and recording site. Six patients had right‐hemispheric coverage.
**Figure S2: Averaged CCEP waveforms.** All CCEP waveforms included in this study, and their averaged response (dark red line) over the range +/− 50 ms surrounding stimulation. Dashed lines highlight the range (−12 to 12 ms) considered at risk of contamination by the stimulation artifact. Ribbon indicates standard deviation of the averaged response.
**Figure S3: All CCEP responses. (Top)** All occipital responses to STG stimulation, across all patients (z‐scored waveforms), ordered by peak latency (for detected responses). N1 responses are marked with a black dot. Vertical white lines indicate (a) the stimulation artifact window (fat line), and (b) the cutoff for N1 definition (80 ms, thin line). **(Bottom)** All occipital responses to Subcentral stimulation.
**Figure S4:** (Top row) Counts of patients for whom at least one response was detected (red) out of total number of patients (grey) for each region and stimulation site. (Remaining rows) Visualization of number of detected responses per patient (red) relative to total number of waveforms (grey). Each bar represents a single patient. Patients are sorted left‐to‐right based on the number of responses detected within each plot.
**Figure S5: (Top)** Average latency of N1 response by patient age, across all STG stimulation/occipital recording sites. (**Bottom**) All N1 responses by patient age, split by stimulation (column) and recording location (row). Different point colors indicate different patients.
**Figure S6:** Time‐frequency representation of all CCEPs from V1/V2 (left), V3/V4 (middle), and V5/hMT+ (right) ROIs. Rows indicate stimulation region. Heatmaps indicate power relative to prestimulus baseline for frequencies up to 50 Hz. The white bar indicates the region of interpolation. Black lines illustrate the standard deviation of the gaussian window used for wavelet generation at each frequency. Single‐trial spectral information was modeled with a mixed‐effects approach to generate fixed‐effect estimates of power relative to baseline for each time/frequency point. For these models (with trial nested within electrode nested within subject), the random effects structure included crossed random effects of electrode, run (for patients with multiple runs), and stimulation pair.
**Figure S7:** Time‐series representation of high‐gamma power for all CCEPs from V1/V2 (left), V3/V4 (middle), and V5/hMT+ (right) ROIs. Rows indicate stimulation region. Lines indicate high‐gamma power relative to prestimulus baseline. The approximate range influenced by interpolation of the stimulation artifact is masked surrounding 0 (+/−1 standard deviation of the gaussian window used for wavelet generation). As in Figure S5, single‐trial spectral information was modeled with a mixed‐effects approach to generate fixed‐effect estimates of power relative to baseline for each time/frequency point. The black line indicates the fixed intercept of the model at a given timepoint, and the ribbons indicate the 95% CI surrounding the fixed effect.

## Data Availability

Data were drawn from openneuro.org/datasets/ds004080. Code necessary to reproduce these results can be found at https://github.com/multisensory‐lab/msl_CCEP_stg2occ.
